# Effects of Elizabethan Collar and Wound Protection Corset on Pain and Discomfort Levels in Cats Undergoing Ovariohysterectomy

**DOI:** 10.1002/vms3.70180

**Published:** 2025-01-10

**Authors:** Ayhan Isil, Latif Emrah Yanmaz

**Affiliations:** ^1^ Department of Surgery, Faculty of Veterinary Medicine Burdur Mehmet Akif Ersoy University Burdur Turkey

**Keywords:** cortisol, feline, pain, scale, stress

## Abstract

**Objective:**

To compare the impacts of Elizabethan collar (EC) and wound protection corset (WPC) on pain and discomfort levels in cats following ovariohysterectomy.

**Study Design:**

Prospective, randomized controlled clinical trial.

**Animals:**

Twenty‐six healthy female cats.

**Methods:**

Animals were randomly assigned to two groups after midline celiotomy: One group wore an EC (*n* = 13), and the other wore a WPC (*n* = 13). Pain was evaluated using the Composite Measure Pain Scale‐Feline (CMPS‐F) and the Multidimensional Pain Scale (MCPS) at intervals of 6, 12, 24, 48, 72 and 96 h, with the requirement for rescue analgesia recorded. Discomfort was assessed through cortisol levels, behavioural observations and the frequency of misbehaviours.

**Results:**

No significant differences were observed between groups at all time intervals for CMPS‐F and MCPS (*p* < 0.05). Rescue analgesic administration was similar between the groups, with 16 administrations in the WPC group and 17 in the EC group (*p* = 0.88). Cortisol levels also showed no significant difference between groups (WPC: 0.05 [0.05–0.3] vs. EC: 0.05 [0.05–0.8]; *p* = 0.09). Behavioural observations revealed significant differences in misbehaviours, with 10 out of 13 cats in the EC group attempting to remove their collars or exhibiting head shaking (*p* < 0.01), whereas no misbehaviours were noted in the WPC group.

**Conclusion:**

Both EC and WPC provided comparable pain management; however, the significant misbehaviours associated with EC use suggest increased discomfort level.

**Clinical Significance:**

The WPC may offer a more comfortable recovery experience for cats following ovariohysterectomy, warranting further investigation.

## Graphical abstract

• The wound protection corset (WPC) and Elizabethan collar (EC) exhibit similar effects on feline pain following ovariohysterectomy.

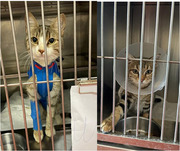

## 1 Introduction

Restraint collars serve as a non‐invasive tool employed by researchers to curb specific behaviours they wish to discourage and offer clinicians a pharmaceutical‐free means of preventing self‐inflicted harm in animals (Jang et al. 2016; Brown 2006a). Among the various restraint collars available, Elizabethan collars (ECs) stand out as the most frequently utilized option. They are employed to curb behaviours such as scratching, the removal of bandages, self‐mutilation at surgical sites, maternal instincts and excessive licking of mammary glands (Gobello et al. 2001; Reuter et al. 2002; Setsu et al. 2021; Martin de Bustamante et al. 2019; Stine, Odum, and Mertens 2018). However, EC restricts certain behaviours in cats, such as their ability to self‐clean by licking and their capacity to scratch their heads (Eckstein and Hart 2000). Furthermore, these collars have the potential to impede peripheral vision and hearing, which can be uncomfortable for cats (Shenoda et al. 2020).

Applying a collar to an animal has the potential to induce a poorer quality of life (Shenoda et al. 2020). Mitigating stress in sick animals is of paramount importance for facilitating accurate diagnosis and treatment (Chávez et al. 2021). Although animals may appear to acclimate to wearing collars with time, a study conducted on rats revealed that the use of EC resulted in decreased feed intake and a reduction in body weight (Brown 2006b). Furthermore, it was observed that there was an immediate negative impact on the animals’ demeanour when they wore EC and an immediate improvement in their demeanour upon collar removal (Shenoda et al. 2020).

The wound protection corset (WPC) is constructed from breathable lycra body suits (Campbell 2006), providing a comfortable and ventilated cover for animals, which may prevent them from harming wounds. Additionally, it incorporates durable polyester materials with minimal shedding properties (Ersoy, Duran, and Tayyar 2015). The aim of this study was to compare the effects of the EC and WPC on pain and discomfort levels in cats after ovariohysterectomy. Our hypothesis is that the WPC may cause less postoperative pain and discomfort in cats compared to the EC. Because gentle compression has been shown to reduce pain by providing support and minimizing discomfort (Sharp 2012), the WPC is believed to alleviate pain by gently compressing and supporting the surgical site, reducing movement and the associated discomfort. Although the EC effectively prevents self‐trauma, its restrictive nature may contribute to increased discomfort.

## 2 Materials and Methods

This prospective, randomized controlled clinical trial was conducted in Burdur Mehmet Akif Ersoy University Animal Hospital, Burdur, from September 2023 to October 2023. Written informed consent was obtained from the owners of the cats planned to undergo ovariohysterectomy before inclusion in the study, and the study protocol was carried out after approval by the Burdur Mehmet Akif Ersoy University Animal Experiments Local Ethics Committee (Decision no: 08.06.2023/1139).

### 2.1 Animals

Animals were considered for inclusion in this study on the basis of comprehensive assessments, including thorough physical examinations, review of medical histories and laboratory tests such as a complete blood count, serum biochemistry profile and blood glucose levels. Eligibility criteria for cats included a temperament score of 1 (Deutsch et al. 2017). Exclusion criteria for this study included animals that were either younger than 1 year or older than 3 years, with weights below 2 kg or exceeding 5 kg. Additionally, animals with an American Society of Anaesthesiologists score other than 1, those exhibiting aggression, showing signs of current pain and inflammation, being unwell or having received analgesic or anti‐inflammatory medications within 14 days prior to the study were excluded. Animals were housed individually in adjacent cages of a cat ward in the animal hospital. Preceding the initiation of the study, cats underwent a 2‐day acclimation period to familiarize them with the observer, study cages and pain assessment tools.

### 2.2 Surgery

Cats were fasted up to 6 h before induction, but no water restriction was applied. They were weighed, and the right forelimbs were shaved for catheterization of the cephalic vein using a 22‐gauge catheter to infuse lactated Ringer's solution at a rate of 3 mL/kg/h throughout the surgery. Intramuscular administration of butorphanol (0.2 mg/kg; Butomidor 10 mg/mL; Richter Pharma, Austria) followed by medetomidine (0.04 mg/kg; Domitor, 1 mg/mL; Orion, Finland) was performed. Anaesthesia induction was achieved using ketamine (10 mg/kg; Ketasol 10%, 10 mL, Interhas Richter Pharma, Wels, Austria) intravenously. After the cats were induced, endotracheal intubation was performed with a 3.5 mm cuffed endotracheal tube. The animals were placed in dorsal recumbency on a warmed blanket. All surgeries were performed by the primary author and one assistant. Hair was clipped at the surgical site, and the skin was prepared for asepsis. A 2.5 cm ventral midline celiotomy incision was made, starting from the caudal border of the umbilicus. Electrosurgical equipment was used to incise the skin and subcutaneous tissue. An initial small incision was made in the linea alba, then extended bilaterally with scissors to access the abdominal cavity. The left and right ovaries, along with the cervix uteri, were gently retracted and doubly ligated using 3‐0 polydioxanone suture, followed by cutting. Round ligaments were carefully severed with scissors, and any small vessels were cauterized if bleeding occurred. Abdominal closure was achieved with a simple continuous 3‐0 polyglactin 910 suture, and the skin was closed using a simple interrupted pattern with 4‐0 nylon suture material (Pereira et al. 2018). The duration of the surgery (measured from the first incision to the placement of the last suture) and the duration of anaesthesia (from induction to extubation) were recorded.

Throughout the surgery, pulse rate (PR) and peripheral capillary oxygen saturation (SpO_2_) were monitored using a pulse oximetry probe positioned on the tongue. Respiratory rate (RR) was assessed by counting chest movements. Rectal temperature (RT) was measured with a rectal probe inserted into the 5 cm rectum. Noninvasive mean arterial pressure (MAP) was recorded using a 2.5 cm wide inflatable cuff placed just proximal to the tarsus. All anaesthesia parameters were documented at 5‐min intervals with a veterinary vital signs monitor (C80‐V Veterinary Multi Parameter Monitor, Comen, China). The cats were extubated when palpebral reflexes returned. At the end of the surgery, intramuscular atipamezole (0.2 mg/kg; Antisedan; 5 mg/mL; Orion, Finland) was injected. Subcutaneous meloxicam (0.2 mg/kg; Metacam, Boehringer Ingelheim, Germany) was administered immediately after surgery and continued for 2 consecutive days.

### 2.3 Groups

**FIGURE 1:**
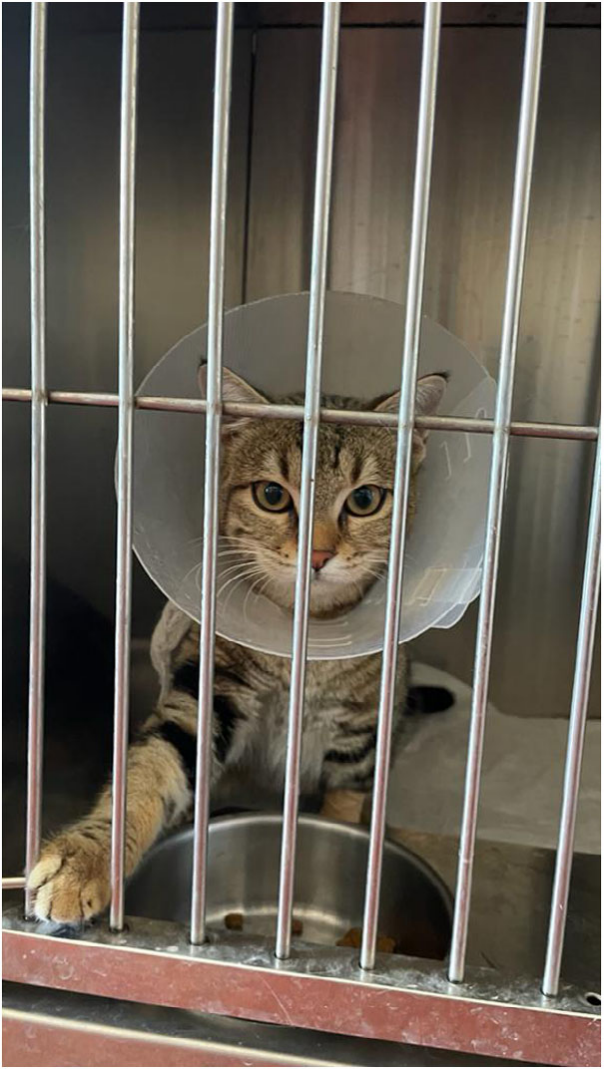
A cat wearing a depth of 10 cm Elizabethan collar.

**FIGURE 2:**
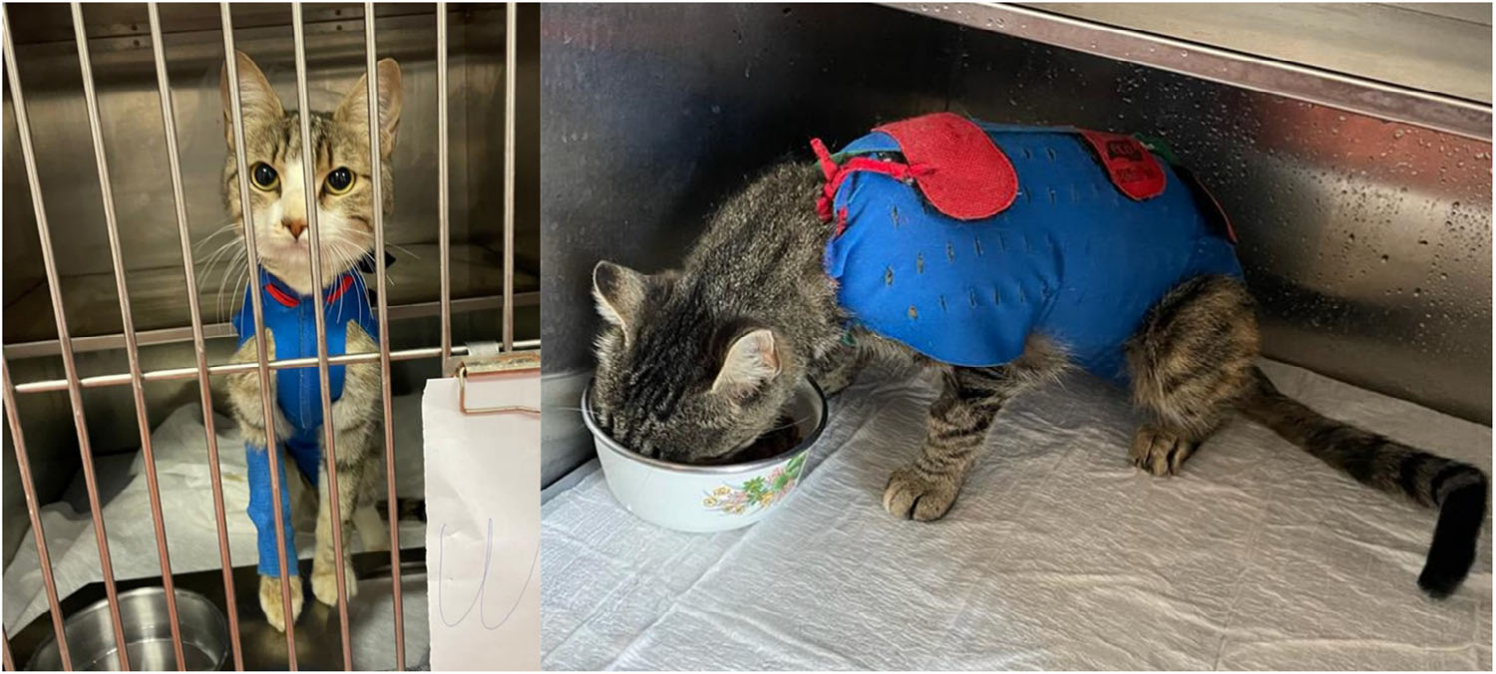
A cat wearing a wound protective corset.

After the surgery, cats were randomly assigned (http://www.randomization.com) to two groups: EC (*n* = 13) and WPC (*n* = 13). The EC group was fitted with a depth of 10 cm EC (Kruuse, Langeskov, Denmark) (Figure 1), whereas the WPC group was outfitted with x‐small sizes of the WPC (Winpet, Nigde, Turkey) (Figure 2).

### 2.4 Pain Evaluation

The cats in both groups were assessed prior to premedication (0) and after surgery at 6, 12, 24, 48, 72 and 96 h, utilizing the UNESP‐Botucatu Multidimensional Composite Pain Scale (MCPS) and the Glasgow Composite Measurement Pain Scale‐Feline (CMPS‐F) (Brondani et al. 2013; Reid et al. 2017). Butorphanol at 0.2 mg/kg was administered intravenously as rescue analgesic when the cat scored higher than or equal to the 6 for (MCPS ≥ 6/27 [without measuring blood pressure]) or 5 for (CMPS‐F ≥ 5/20). The same person recorded the pain scores for all measurements.

### 2.5 Discomfort Evaluation

A saliva was collected with tooth cleaning sponges into eppendorf tubes with a volume of 0.1 mL at 0, 6, 12, 24, 48, 72 and 96 h. The samples were kept at −80°C until the procedure was performed. The cortisol levels in all saliva samples were measured by an immunoassay (Salivary Cortisol ELISA Kit—Salimetrics, California, USA Samples) for analysing cortisol levels. Salivary cortisol samples were collected at consistent times to control for circadian variations, providing a physiological measure of discomfort. Behavioural observations were conducted to identify signs of discomfort, such as excessive grooming, vocalization and restlessness, while also monitoring for misbehaviours, including attempts to remove the EC or WPC, head shaking and inactivity. The frequency and intensity of these behaviours were recorded to assess any changes over time.

### 2.6 Statistical Analysis

A sample size was determined to detect a clinically important target difference of 10% in MCPS between treatments, with a significance level (α) of 0.05 and a statistical power of 90% (*β* = 0.10). On the basis of a standard deviation of 0.5 from a previous study evaluating the analgesic efficacy of intraperitoneal bupivacaine in cats undergoing ovariohysterectomy (Benito et al. 2016), the calculation indicated that 13 cats were needed per group.

The data obtained from the groups were analysed using SPSS 23.0 (SPSS Inc., Chicago, IL, USA). The significance level used was accepted as *p* < 0.05. The Shapiro–Wilk test was used to assess the normality of the data distribution, and visual assessments, including histograms and dot plots, were also performed to confirm the distribution. Changes of pain scores over time within groups were evaluated using the Friedman test followed by Dunn's test. Salivary cortisol data were analysed using the Kruskal–Wallis test to compare cortisol levels across multiple time points, followed by Mann–Whitney *U* tests for pairwise comparisons between specific time points. The number of cats requiring rescue analgesia and the occurrence of misbehaviours between the two groups were compared using Fisher's exact test. Data were reported as mean ± standard deviation for normally distributed variables and as median (range) for non‐normally distributed variables.

## 3 Results

Table 1 shows body condition score, body weight, haematocrit, alanine aminotransferase, aspartate aminotransferase, blood urea nitrogen, glucose and total protein. No anaesthetic‐related complications were observed throughout the study. The duration of surgery was 24.92 ± 5.33 min in the EC group and 25.85 ± 6.23 min in the WPC group. The duration of anaesthesia was 41 min (range: 28–70) in the EC group and 40 min (range: 30–80) in the WPC group.

**TABLE 1:** Body condition score (BCS), body weight (kg), haematocrit (%), alanine aminotransferase (U/L), aspartate aminotransferase (U/L), blood urea nitrogen (mg/dL), glucose (mg/dL) and total protein (g/dL) in 26 cats undergoing midline celiotomy, either wearing Elizabethan collar (EC) or wound protection corset (WPC).

Variables	Groups	
	EC (*n* = 13)	WPC (*n* = 13)
BCS (1–9)	5 (4–6)	5 (4–6)
Body weight (kg)	2.82 ± 0.59	3.10 ± 0.84
Haematocrit (%)	37.25 ± 6.22	38.75 ± 4.76
Alanine aminotransferase (U/L)	52.15 ± 14.98	59.29 ± 23.45
Aspartate aminotransferase (U/L)	23.23 ± 6.58	25.31 ± 5.92
Blood urea nitrogen (mg/dL)	23.46 ± 2.7	23.38 ± 3.1
Glucose (mg/dL)	89.83 ± 20.42	91.51 ± 22.79
Total protein (g/dL)	6.94 ± 0.6	6.97 ± 0.53

*Note*: Data are presented as mean ± standard deviation or median (range).

### 3.1 Pain Evaluation

No significant differences were noted between groups at all time intervals concerning CMPS‐F and MCPS. Specifically, at 96 h, there were no significant differences compared to baseline levels for both CMPS‐F (1 [0–3]; *p* = 0.09) and MCPS (2 [0–4]; *p* = 0.19) in the WPC group (Table 2). Overall, the WPC group required rescue analgesic administration 16 times, whereas the EC group required it 17 times; however, these frequencies were not significantly different (*p* = 0.88) (Table 3).

**TABLE 2:** UNESP‐Botucatu Multidimensional Composite Pain Scale (MCPS), Glasgow Composite Measurement Pain Scale (CMPS‐F), and salivary cortisol levels in animals fitted with either a wound protection corset (WPC) or an Elizabethan collar (EC).

Time point (h)								
Variable	Group	*T*0	*T*6	*T*12	*T*24	*T*48	*T*72	*T*96
CMPS‐F	EC	0 (0–2)	6 (4–8)	5 (4–8)	5 (3–7)	4 (2–6)	3 (1–5)	2 (0–4)
	WPC	1 (0–2)	5 (3–7)	4 (3–6)	4 (2–6)	3 (2–5)	2 (1–4)	1 (0–3)^*^
*p*		0.76	0.33	0.27	0.28	0.67	0.92	0.24
MCPS	EC	1 (0–3)	8 (5–11)	7 (5–10)	7 (3–11)	5 (2–8)	3 (1–6)	3 (1–5)
	WPC	1 (0–3)	8 (6–9)	7 (5–8)	7 (4–8)	5 (3–7)	4 (1–5)	2 (0–4)^†^
*p*		0.67	0.44	0.52	0.36	0.90	0.79	0.38
Salivary cortisol (µg/dL)	EC	0.05 (0.05–0.21)	0.05 (0.05–0.10)	0.05 (0.05–0.20)	0.05 (0.05–0.20)	0.05 (0.05–0.07)	0.05 (0.05–0.10)	0.05 (0.05–0.10)
	WPC	0.05 (0.05–0.11)	0.05 (0.05–0.20)	0.05 (0.05–0.15)	0.05 (0.05–0.27)	0.05 (0.05–0.19)	0.05 (0.05–0.10)	0.05 (0.05–0.10)
*p*		0.78	0.71	0.69	0.93	0.54	0.65	0.36

*Note*: No significant difference compared to baseline (*p = 0.09; †*p* = 0.19). Measurements were taken at baseline (*T*0) and at 6 (*T*6), 12 (*T*12), 24 (*T*24), 48 (*T*48), 72 (*T*72) and 96 h (*T*96) postoperatively. Data are presented as median (range).

**TABLE 3:** The number of cats that received rescue analgesia (butorphanol 0.2 mg/kg intravenously) when the pain score was equal to or higher than 6 on the UNESP‐Botucatu Multidimensional Composite Pain Scale (MCPS ≥ 6/27) or 5 on the Glasgow Composite Measurement Pain Scale (CMPS‐F ≥ 5/20).

		Time point (h)						
Variable	Group	*T*6	*T*12	*T*24	*T*48	*T*72	*T*96	Total
CMPS‐F	EC	4	3	3	2	1	0	13/78 (16.6%)
	WPC	4	3	3	1	0	0	11/78 (14.1%)
MCPS	EC	4	5	4	3	1	0	17/78 (21.8%)
	WPC	4	4	4	2	0	0	16/78 (20.5%)

*Note*: Pain was assessed at 6 (*T*6), 12 (*T*12), 24 (*T*24), 48 (*T*48), 72 (*T*72) and 96 h (*T*96) postoperatively.

Abbreviations: EC, Elizabethan collar; WPC, wound protection corset.

### 3.2 Discomfort Evaluation

The cortisol levels exhibited no significant difference between WPC (0.05 [0.05–0.3]) and EC (0.05 [0.05–0.8]) groups (*p* = 0.09). No excessive grooming, vocalization or restlessness was observed in either group throughout the study period. In the EC group, 3 cats (3/13) attempted to remove their EC using their paws, and 7 cats (7/13) exhibited head shaking, resulting in a total of 10 out of 13 cats displaying misbehaviours. In contrast, no misbehaviours were observed in the WPC group (0/13). The difference in the frequency of misbehaviours between the EC and WPC groups was statistically significant (*p* < 0.01). The frequency of these misbehaviours in the EC group remained consistent over time, whereas no behaviours were noted in the WPC group at any observation point.

## 4 Discussion

The aim of this study was to compare the effects of the EC and WPC on postoperative pain and discomfort in cats after ovariohysterectomy. Notably, this is the first investigation examining the impact of WPC and EC on both pain and discomfort during feline postoperative recovery. We used a combination of pain scores, behavioural observations and salivary cortisol levels to assess recovery. Our hypothesis, suggesting that the WPC may reduce postoperative pain and discomfort compared to the EC, was partially supported. Although statistical analysis showed no significant differences in pain scores between the WPC and EC groups at various time points, the absence of significant differences in the WPC group at 96 h compared to baseline levels may suggest a trend towards reduced long‐term pain in the WPC group. Regarding discomfort, no excessive grooming, vocalization or restlessness was observed in either group, and cortisol levels were comparable, indicating similar levels of discomfort between the groups. These findings underscore the need for further research to explore the potential benefits of the WPC in feline postoperative recovery.

We hypothesized that the WPC might alleviate postoperative pain and reduce discomfort due to its design, which offers a gentler and more flexible approach. The EC, with its rigid structure, is known to cause discomfort by limiting natural behaviours such as grooming and movement (Shenoda et al. 2020). In contrast, the WPC imposes less physical restriction, allowing more natural movement, which may reduce the discomfort associated with restricted mobility. Additionally, because the WPC does not obstruct vision or cause mechanical irritation around the neck and head—as evidenced by observations of head shaking and attempts to remove the EC in some cats—it may result in less agitation and, indirectly, lower overall discomfort. Although the precise mechanisms remain speculative, reducing external sources of discomfort, such as physical restriction and visual obstruction, could contribute to a more comfortable postoperative recovery for cats. These interpretations warrant further validation through studies specifically designed to isolate and quantify these effects.

Ovariohysterectomy, which requires a midline or lateral celiotomy, is frequently performed surgical procedure in veterinary practice (Oliveira et al. 2014). Even though the application selected for the study may not seem relevant to all clinical cases where protection would be required, ovariohysterectomy serves as a relevant and practical model for evaluating the efficacy of EC and WPC on pain and discomfort levels in cats undergoing midline celiotomy. By standardizing incisions between groups, this model allows for a direct comparison of the protective devices’ effects, thereby enhancing the validity and applicability of our study findings.

ECs frequently restrict the visual field, may impede peripheral vision and hearing and can evoke fear and discomfort in cats (Shenoda et al. 2020). Similarly, in the current study, in the EC group, three cats attempted to remove their EC using their paws, and seven cats exhibited head shaking. In contrast, no such behaviours were observed in the WPC group. The absence of an external device like the EC in the WPC group did not provoke similar reactions. Additionally, no signs of excessive grooming, vocalization, restlessness, head shaking or inactivity were observed in the WPC group, suggesting that the discomfort associated with the EC was more pronounced. This may attribute to our hypothesis that WPC is more comfortable for cats than EC.

CMPS‐F and MCPS are commonly used tools to evaluate acute pain following surgery (Steagall and Monteiro 2019), whereas stress can be assessed through plasma and serum cortisol levels (Iki et al. 2011; Mazzotti and Boere 2009). However, it is noteworthy that the salivary cortisol values in our study were consistently lower than the reference values established for felines (Da Silva et al. 2017). One possible explanation is the lack of species‐specific validation for the immunoassay used, which may have affected the accuracy of cortisol detection in cats. A previous study in dogs also found salivary cortisol levels to be lower than expected (Bowman et al. 2015), further suggesting potential species differences in cortisol expression or assay sensitivity.

The frequency of rescue analgesia was extensive, suggesting that the analgesia protocol may not have been fully adequate in managing postoperative pain. This could potentially affect discomfort levels, as poorly controlled pain can contribute to increased discomfort. However, despite the high frequency of rescue analgesia, the salivary cortisol levels remained consistently below the reference range. One possible explanation for this discrepancy is that the salivary cortisol levels may not have been sensitive enough to detect acute discomfort caused by pain in this context, as cortisol levels can fluctuate based on individual physiological responses and external influences, such as the hospital environment. Although rescue analgesia was frequently administered, the consistently low cortisol levels observed may not fully reflect the animals’ pain or discomfort levels. It is possible that other mechanisms, such as individual variability in cortisol response or the influence of factors unrelated to pain, contributed to these findings. Additionally, cortisol alone may not provide a complete picture of discomfort, as it can be influenced by a variety of factors. Therefore, incorporating other physiological or behavioural indicators of discomfort could offer a more comprehensive understanding of the cats’ postoperative recovery.

The absence of a discernible difference in the requirement for rescue analgesia between the groups in our study suggests a comparable level of pain for cats during the acute phase of both treatments. A prior study noted that, despite the similarity in the need for rescue analgesia between the CMPS‐F and MCPS scales, distinctions might exist between the two scales regarding the necessity for additional analgesic intervention (Steagall et al. 2018). Likewise, in this study, although the two scales exhibited similarities, an examination of rescue analgesics revealed a higher demand in the MCPS group. The efficiency of CMPS‐F may render it a more favourable choice over MCPS due to its quicker application and user‐friendly nature (Steagall and Monteiro 2019). Nevertheless, opting for MCPS in the assessment of rescue analgesia yields a more thorough evaluation of pain.

The primary limitation of this study was the lack of long‐term discomfort level measurements, as the assessment spanned only 96 h. An environmental factor that could have influenced discomfort levels during the study was the variability in conditions within the cat ward of the animal hospital. Fluctuations in noise levels, the presence of other cats and general ward activity may have contributed to differences in discomfort at various time points. However, these potential influences were not specifically controlled for. This limitation highlights the need for caution when interpreting the discomfort‐related findings. Additionally, conducting the study in the cat ward of an animal hospital may have introduced stressors not present in home settings, where cats might experience more freedom and less stress (Hirsch 2016). This difference in environment could have provided a more accurate assessment of discomfort levels. Another limitation was the absence of functional assessments, such as owners’ questionnaires or accelerometer data, which would have facilitated the evaluation of the cats’ comfort levels with each device. The lack of body weight and food intake data limited the ability to assess the full impact of the EC and WPC on the cats’ overall well‐being. Including these measurements could have provided a more comprehensive understanding of the devices’ effects. Furthermore, other stress factors, including blood cortisol, as blood glucose, TNF‐α, interleukin 1 beta and interleukin 6 levels, could have been used (Hudec and Griffin 2020; Stella, Croney, and Buffington 2013); however, these evaluations need blood analysis, which were not accepted by owners due to blood samples taken various times.

In conclusion, this study demonstrated that both the EC and WPC were associated with comparable levels of pain management, as indicated by similar pain scores and cortisol levels during the postoperative period. Although neither device provided direct analgesia, their impact on postoperative recovery behaviours differed significantly. Cats in the EC group displayed more frequent attempts to remove their collars and exhibited head shaking, suggesting increased discomfort. In contrast, the absence of these behaviours in the WPC group suggests that this device may offer a more comfortable recovery experience for cats following ovariohysterectomy. These findings highlight the potential of the WPC as a viable alternative to the EC in veterinary postoperative care, particularly for improving comfort.

## Author Contributions

**Ayhan Isil**: study design, data management, data interpretation, preparation and revision of the manuscript. **Latif Emrah Yanmaz**: data management, data interpretation, statistical analysis and revision of the manuscript.

## Ethics Statement

The study protocol was carried out after approval by the Burdur Mehmet Akif Ersoy Local Ethics Committee (Decision no: 08.06.2023/1139).

## Conflicts of Interest

The authors declare no conflicts of interest.

### Peer Review

The peer review history for this article is available at https://publons.com/publon/10.1002/vms3.70180.

## Data Availability

Data available on request from the authors.
